# Comparative in vitro screening identifies beta-hairpin antimicrobial peptide leads against *Piscirickettsia salmonis*, supported by membrane-level molecular dynamics

**DOI:** 10.1186/s12866-026-05304-0

**Published:** 2026-06-17

**Authors:** Jorge F. Beltrán, Jorge G. Farías, Aloyma Lugo, Luis Jimenez, Lisandra Herrera Belén, Sandra N. Flores-Martin, Juan-Alejandro Norambuena, Alejandro J. Yáñez

**Affiliations:** 1 Department of Chemical Engineering, Faculty of Engineering and Science, Universidad de La Frontera, Ave. Francisco Salazar 01145, Temuco, 4811230 Chile; 2 Departamento de Investigación y Desarrollo, Greenvolution SpA, Puerto Varas, Región de Los Lagos Chile; 3 Departamento de Ciencias Básicas, Facultad de Ciencias, Universidad Santo Tomás, Temuco, 4780000 Chile; 4 Centro de Investigación Austral Biotech, Facultad de Ciencias, Universidad Santo Tomas, Avenida Ejército 146, Santiago, 8320000 Chile; 5 Keybio Solution E.I.R.L., Valdivia, Chile; 6 BioChemIntelli Platform, R&D, Temuco, Chile

**Keywords:** *Piscirickettsia salmonis*, Antimicrobial peptides, Tachyplesin I, Protegrin-1, Salmonid rickettsial septicemia, Molecular dynamics

## Abstract

**Background:**

Salmonid rickettsial septicemia caused by *Piscirickettsia salmonis* remains a major driver of antimicrobial use in Chilean aquaculture. Antimicrobial peptides are plausible alternatives, but their activity may depend on peptide scaffold and on the unusual membrane composition predicted for *P. salmonis*.

**Results:**

We screened 13 compounds against *P. salmonis* LF-89 at 16.5 °C over a 7-day incubation period, including 11 cationic antimicrobial peptides together with two non-peptidic reference compounds, florfenicol and chlorhexidine. The assay was performed with three independent biological replicates initiated from separately seeded LF-89 cultures. Because the readout quantified percent inhibition of net OD600 growth rather than conventional complete visible growth inhibition, activity is reported as IC80 and IC90 growth-inhibition endpoints. Among the peptides, the two beta-hairpin peptides in the panel showed the strongest activity. Tachyplesin I showed IC80 and IC90 values of 2 and 16 µg/mL, respectively, whereas Protegrin-1 reached both endpoints at 32 µg/mL. Among the linear peptides, the strongest responses were observed for SMAP-29 and Cecropin B, both with IC80 values of 64 µg/mL. To examine whether the two most active peptide leads showed distinct membrane-interaction behavior, we performed three independent 300 ns all-atom molecular dynamics trajectories per peptide on a phosphatidylethanolamine-biased bilayer. Across replicate trajectories, both peptides adsorbed to the membrane and showed distinct interfacial behavior: Tachyplesin I remained more conformationally compact, whereas Protegrin-1 formed more peptide-lipid contacts and hydrogen bonds, underscoring that monomeric adsorption metrics and growth-inhibition potency are not interchangeable.

**Conclusions:**

Within this 13-compound panel, disulfide-stabilized beta-hairpin peptides showed the strongest growth-inhibitory activity against *P. salmonis*. The molecular dynamics results support scaffold-dependent differences in membrane interaction and prioritize Tachyplesin I and Protegrin-1 as membrane-active leads for follow-up studies against this pathogen. Together, these data provide a robust comparative screening and replicate MD analysis that identifies Tachyplesin I and Protegrin-1 as the leading beta-hairpin candidates in this panel; dedicated safety, serum-stability, host-cell selectivity, and in vivo efficacy studies are warranted as the next stage of translational evaluation.

**Graphical Abstract:**

**Figure:**
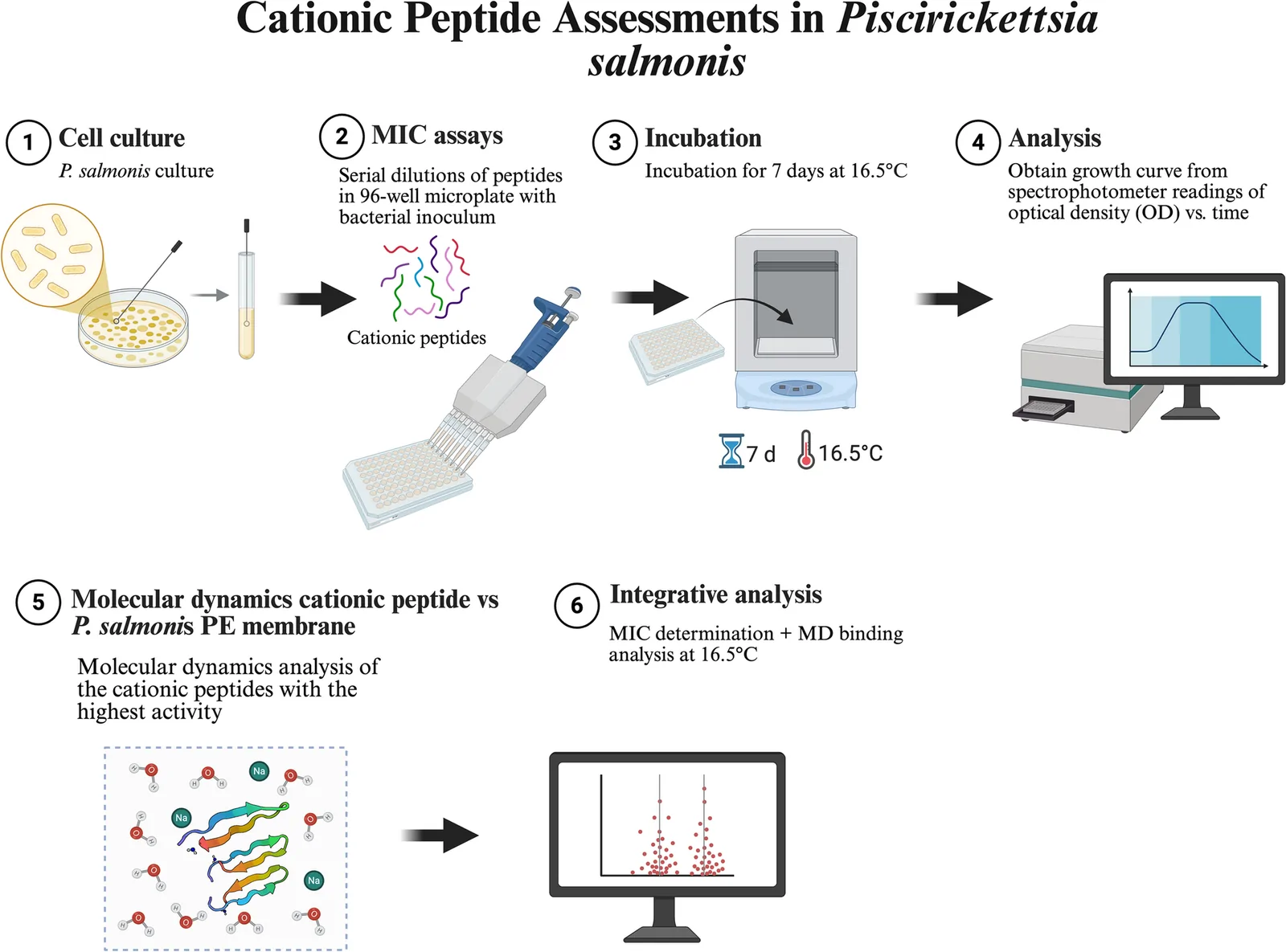

**Supplementary Information:**

The online version contains supplementary material available at https://doi.org/10.1186/s12866-026-05304-0.

## Background

Salmonid rickettsial septicemia (SRS), caused by the Gram-negative facultative intracellular bacterium *Piscirickettsia salmonis*, remains one of the most important infectious constraints in Chilean salmon farming [1–4]. The disease causes substantial economic losses and continues to drive antimicrobial use in seawater production systems [1–4]. Current therapeutic interventions rely heavily on florfenicol, with oxytetracycline playing a secondary role [3, 4]. This dependence has sustained broader One Health concerns because aquaculture environments can facilitate the selection and dissemination of antimicrobial resistance genes [4, 5], and *P. salmonis* field isolates with reduced susceptibility patterns have already been described [6].

Cationic antimicrobial peptides (AMPs) are therefore attractive as alternative or complementary anti-infective agents. Many AMPs act primarily through interactions with the bacterial envelope rather than a single intracellular protein target [7–9], and they comprise a large and structurally diverse class of innate defense molecules [8, 10]. Their activity, however, depends strongly on peptide scaffold and membrane context. Alpha-helical linear peptides such as magainins acquire amphipathic structure upon membrane contact and tend to perform better in bilayers enriched in anionic phospholipids [11], whereas beta-hairpin peptides such as tachyplesins and protegrins are disulfide-constrained and preorganized for membrane interaction [12]. Whether these scaffold-dependent differences become especially important in bacteria with atypical envelope composition remains poorly defined.

*P. salmonis* is a useful system in which to examine that question. Genome-scale reconstruction of strain LF-89 identified missing cardiolipin and phosphatidylglycerol biosynthetic capacity, which motivated the phosphatidylethanolamine-biased (PE-biased) inner-membrane model used here [13]. This genome-informed model differs from the canonical Gram-negative membrane represented by *Escherichia coli* [14]. In parallel, structural analysis of *P. salmonis* lipid A identified 4-amino-4-deoxy-L-arabinose (Ara4N) substitutions [15], a modification linked to reduced outer-membrane negative charge and lower susceptibility to cationic compounds [16], and intact lipopolysaccharide structure contributes to virulence in salmon [17]. Together, these observations suggest that *P. salmonis* may present an envelope context different from standard Gram-negative reference systems.

Despite the importance of SRS, efforts to expand the therapeutic repertoire against *P. salmonis* have focused mainly on plant-derived preparations such as *Quillaja saponaria* extracts [18] and target-directed small-molecule discovery campaigns [19], rather than systematic comparisons of structurally distinct AMPs under pathogen-relevant culture conditions.

We therefore asked whether a genome-informed PE-biased membrane model of *P. salmonis* is associated with differential susceptibility to AMP structural classes. To address this question, we screened 13 compounds against *P. salmonis*, comparing beta-hairpin and alpha-helical AMPs alongside florfenicol and chlorhexidine reference compounds. We then performed replicate all-atom molecular dynamics (MD) simulations of the two most active peptides on a PE-biased bilayer to determine whether the experimentally identified leads were accompanied by distinct membrane interaction modes.

## Methods

### Compounds

Thirteen compounds were evaluated in this study. The peptide panel was selected to compare commercially available, high-purity cationic AMP scaffolds with documented antimicrobial activity and structural diversity. Eleven cationic AMPs representing two major structural classes were included: two disulfide-stabilized beta-hairpin peptides (Tachyplesin I, HY-P1632; Protegrin-1, HY-P1633) and nine linear peptides described as alpha-helical or amphipathic linear/hybrid AMPs (SMAP-29, HY-P2460; Cecropin A(1–7)-Melittin A(2–9), HY-P3914; Cecropin A, HY-P1539; Cecropin B, HY-P0092; Dermaseptin-S1, HY-P5594; Dermaseptin, HY-P0263; Temporin L, HY-P2523; Magainin 2, HY-P0270; Cecropin P1, HY-P2317). In addition, the panel included florfenicol, the standard-of-care antibiotic for SRS in Chilean aquaculture, and chlorhexidine, a membrane-active biguanide antiseptic. The antimicrobial peptides were obtained from MedChemExpress (MCE) as lyophilized powders with purity greater than or equal to 95%. Florfenicol and chlorhexidine were included as non-peptidic reference compounds. Peptide identities, catalog codes, structural classes, approximate net charges, and molecular masses are listed in Table 1.

**Table 1:** Peptide sequences, physicochemical properties, and growth-inhibition endpoints against *P. salmonis* LF-89. IC80 and IC90 denote the lowest tested concentrations producing at least 80% and 90% inhibition of net OD600 growth at day 7, respectively; they are not conventional clinical MIC values

Peptide	Catalog code	Structural class	Sequence	Approx. charge	MW (Da)	IC80, µg/mL (µM)	IC90, µg/mL (µM)	Inhibition at 128 µg/mL (%)
Tachyplesin I	HY-P1632	Beta-hairpin, 2 disulfides	KWCFRVCYRGICYRRCR-NH_2_ Cys_3_-Cys_16_, Cys_7_-Cys_12_	+ 7	2263.74	2 (0.9)	16 (7.1)	86.7
Protegrin-1	HY-P1633	Beta-hairpin, 2 disulfides	RGGRLCYCRRRFCVCVGR-NH_2_ Cys_6_-Cys_15_, Cys_8_-Cys_13_	+ 7	2155.61	32 (14.8)	32 (14.8)	99.5
SMAP-29	HY-P2460	Linear cathelicidin, alpha-helical	RGLRRLGRKIAHGVKKYGPTVLRI IRIAG	+ 10	3256.02	64 (19.7)	64 (19.7)	99.7
Cecropin B	HY-P0092	Linear alpha-helical	KWKVFKKIEKMGRNIRNGIVKAGP AIAVLGEAKAL-NH_2_	+ 7	3834.67	64 (16.7)	128 (33.4)	98.6
Cecropin A	HY-P1539	Linear alpha-helical	KWKLFKKIEKVGQNIRDGIIKAGP AVAVVGQATQIAK-NH_2_	+ 7	4003.78	128 (32.0)	128 (32.0)	104.8
Cecropin P1	HY-P2317	Linear alpha-helical	SWLSKTAKKLENSAKKRISEGIAI AIQGGPR	+ 5	3338.86	128 (38.3)	128 (38.3)	98.6
Cecropin A (1–7)-Melittin A (2–9)	HY-P3914	Linear hybrid peptide	KWKLFKKIGAVLKVL-NH_2_	+ 6	1770.30	128 (72.3)	128 (72.3)	100.6
Dermaseptin	HY-P0263	Linear alpha-helical	ALWKTMLKKLGTMALHAGKAALGA AADTISQGTQ	+ 4	3455.10	128 (37.0)	128 (37.0)	106.8
Dermaseptin-S1	HY-P5594	Linear alpha-helical	ALWKTMLKKLGTMALHAGKAALGA AADTISQGTQ	+ 4	3455.06	> 128 (> 37.0)	> 128 (> 37.0)	37.0
Magainin 2	HY-P0270	Linear alpha-helical	GIGKFLHSAKKFGKAFVGEIMNS	+ 3	2466.90	128 (51.9)	128 (51.9)	119.4
Temporin L	HY-P2523	Short linear alpha-helical	FVQWFSKFLGRIL-NH_2_	+ 3	1639.98	128 (78.0)	128 (78.0)	120.8

Approximate charge values are reported at neutral pH and may vary slightly depending on terminal ionization and histidine protonation assumptions. Micromolar values were calculated from the mass concentration and molecular weight as µM = (µg/mL x 1000)/MW. Dermaseptin and Dermaseptin-S1 were tested as separate purchased catalog products, although the vendor-reported mature peptide sequence is the same

### Bacterial strain and culture conditions

An ATCC strain of *Piscirickettsia salmonis* LF-89 (ATCC VR-1361) was used throughout this study. Bacteria were cultured in Austral liquid medium [20] at 16.5 °C, a pathogen-relevant temperature close to the reported optimal replication range of *P. salmonis*, and, following the previously described inhibition workflow, cultures were grown prior to assay setup to an optical density at 600 nm (OD600) of 0.85 and the inoculum used for microplate assays was adjusted to an OD600 of 0.1 [19].

### Broth microdilution growth-inhibition assay

The antimicrobial activity of each compound was assessed by broth microdilution in 96-well microplates, following the general *P. salmonis* inhibition assay framework described previously [19]. Compounds were tested at ten two-fold serial dilutions ranging from 128 to 0.25 µg/mL, with untreated growth controls, sterile controls, and medium-only blanks included on each plate. Assays were performed using three independent biological replicates. Each replicate was initiated from a separately seeded *P. salmonis* LF-89 culture prepared from the ATCC strain and processed independently through the 7-day incubation. OD600 was recorded daily from day 0 (d0, immediately after inoculation) through day 7 (d7) using a Synergy 2 microplate reader (BioTek) operated with Gen5 v2.07 software.

### Data processing and IC80/IC90 determination

Raw OD600 values were corrected by subtracting the mean absorbance of the medium-only blank wells for the corresponding concentration and time point. Net growth for each well was calculated as the blank-corrected OD600 at d7 minus the blank-corrected OD600 at d0. Percent inhibition was computed as (1 minus net growth of treated well divided by net growth of growth control) multiplied by 100. Values exceeding 100% indicate that the blank-corrected endpoint OD600 was lower than the starting OD600 for that condition; because no colony-forming unit or viable-count assay was performed, these values were not interpreted as evidence of reduced viable counts. The IC80 and IC90 growth-inhibition endpoints were defined as the lowest concentrations achieving at least 80% and 90% inhibition of net OD600 growth, respectively, relative to the untreated growth control at d7. All antimicrobial data processing and IC80/IC90 determination were performed in R version 4.5.0 [21].

### Growth curve analysis

Growth curves were generated for each compound by plotting the mean blank-corrected OD600 across the three biological replicates at each concentration for all eight time points (d0 through d7). Error bars represent the standard deviation of the biological replicates. The untreated growth control curve was included on each plot for reference. Growth-curve visualization was generated programmatically in the same R workflow.

### Molecular dynamics simulations

All-atom MD simulations were performed for the two beta-hairpin peptides that showed the highest activity among the peptides tested: Tachyplesin I and Protegrin-1. Initial peptide coordinates were obtained from the Protein Data Bank: Tachyplesin I from PDB entry 1WO0, an NMR solution structure in water [22]; and Protegrin-1 from PDB entry 1ZY6, an NMR structure determined in lipid bilayers [23]. For Protegrin-1, only chain A of the dimeric structure was retained for simulation.

Simulation systems were assembled using CHARMM-GUI Membrane Builder [24]. Each system consisted of a single peptide positioned approximately 20 Å above the center of a symmetric lipid bilayer containing 128 lipids per leaflet (90 POPE and 38 DOPE per leaflet, corresponding to a 70:30 molar ratio). This PE-biased composition was chosen to approximate a genome-informed inner-membrane model for *P. salmonis*, based on the absence of phosphatidylglycerol and cardiolipin biosynthetic capacity in the reconstructed network [13]. The systems were solvated with TIP3P water molecules with a minimum padding of 25 Å and neutralized with 150 mM NaCl. Protein parameters were described with CHARMM36m [25] and lipid parameters with the CHARMM36 lipid force field [26]. Hydrogen mass repartitioning (HMR) was applied to allow a 4 fs integration time step [27].

Simulations were performed using OpenMM v8.5 [28] with the OpenCL platform on an Apple M3 Max GPU (40 cores; 128 GB unified memory). Each system contained approximately 81,000 atoms. The equilibration protocol followed the six-stage CHARMM-GUI scheme with progressively decreasing positional restraints on lipid and peptide heavy atoms. For each peptide, production was carried out as three 300 ns trajectories in the NPT ensemble at 289.65 K (16.5 °C) using a Langevin integrator with a friction coefficient of 1 ps⁻¹ and a Monte Carlo membrane barostat at 1 bar. The original trajectory was retained as replicate 1, and two additional production trajectories were generated from the equilibrated system with independently assigned initial velocities using distinct random seeds. For file management, each 300 ns trajectory was saved as 30 consecutive 10 ns production segments, with each segment restarted from the final state of the preceding segment; these 10 ns segments were not treated as independent replicas. Nonbonded interactions were computed using the particle mesh Ewald (PME) method with a real-space cutoff of 12 Å and a force-switch function applied between 10 and 12 Å for van der Waals interactions. Coordinates were saved every 100 ps, yielding 3,000 frames per trajectory and 9,000 analyzed frames per peptide. Full system composition, simulation parameters, and setup snapshots are provided as supplementary material.

### Trajectory analysis

Trajectory analysis was performed using MDAnalysis v2.10 [29, 30]. The following properties were computed for each simulation: (i) backbone root-mean-square deviation (RMSD) relative to the initial structure after least-squares superposition, as a measure of peptide structural stability; (ii) the signed z-displacement between the peptide center of mass and the mean z-coordinate of the upper-leaflet phosphorus atoms, as a measure of peptide-membrane proximity; (iii) the number of atomic contacts within 4 Å between peptide and lipid atoms at each frame, using minimum-image distances under periodic boundary conditions; the center-of-mass z-displacement and residue-depth values were corrected using the minimum-image displacement in z to avoid periodic wrapping artifacts; (iv) per-residue insertion depth relative to the upper-leaflet phosphate plane, evaluated at seven approximately equally spaced time points (0, 50, 100, 150, 200, 250, and 300 ns); and (v) the number of hydrogen bonds between peptide donor atoms (backbone and side-chain nitrogen atoms) and lipid acceptor atoms (phosphate and glycerol oxygen atoms of POPE and DOPE), using a donor-acceptor distance cutoff of 3.5 Å and an angle cutoff of 150 degrees. All analysis scripts used in this study are archived in the project workspace and are available from the corresponding author on reasonable request.

## Results

### Beta-hairpin peptides show the strongest growth-inhibitory activity among the tested AMPs

The growth-inhibition screen revealed a clear activity ranking across the AMP panel. Among the peptides, the two disulfide-stabilized beta-hairpin scaffolds were the most active: Tachyplesin I showed IC80 and IC90 values of 2 and 16 µg/mL, respectively, whereas Protegrin-1 reached both thresholds at 32 µg/mL. Among the linear alpha-helical peptides, the strongest responses were observed for SMAP-29 and Cecropin B, both of which reached IC80 at 64 µg/mL, while most of the remaining linear peptides required 128 µg/mL or failed to achieve IC80 within the concentration range tested. Among the non-peptidic reference compounds, florfenicol showed the lowest endpoint values overall with IC80 and IC90 values of 0.5 µg/mL, whereas chlorhexidine showed IC80 and IC90 values of 4 µg/mL. Dermaseptin-S1 showed no measurable IC80 or IC90 endpoint by day 7. These data established an experimental ranking for subsequent membrane-interaction analysis. Table 1 summarizes the peptide sequences, key physicochemical properties, and growth-inhibition endpoints for the panel.

**Fig. 1:**
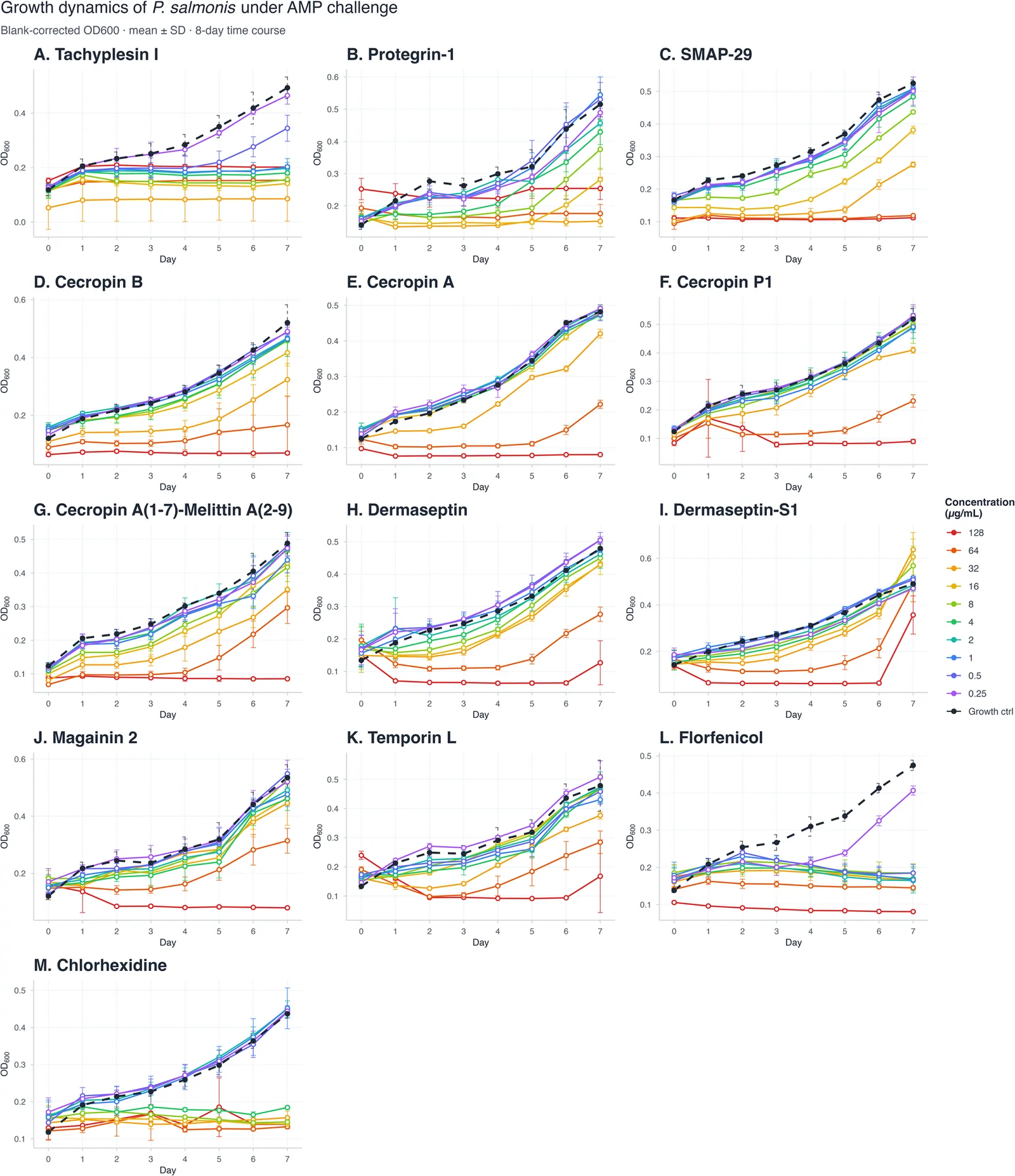
Growth curves of the 13-compound panel against *P. salmonis* over 7 days. Curves show mean blank-corrected optical density at 600 nm (OD600) across three biological replicates for each concentration at 16.5 °C, together with the untreated growth control; error bars show standard deviation. **A** Tachyplesin I. **B** Protegrin-1. **C** SMAP-29. **D** Cecropin B. **E** Cecropin A. **F** Cecropin P1. **G** Cecropin A (1–7)-Melittin A (2–9). **H** Dermaseptin. **I** Dermaseptin-S1. **J** Magainin 2. **K** Temporin L. **L** Florfenicol. **M** Chlorhexidine

This ranking is also evident in the full growth-curve panel shown in Fig. 1. Tachyplesin I and Protegrin-1 (Fig. 1A, B) maintained the broadest sustained suppression of bacterial growth across the tested concentration range, whereas SMAP-29 and Cecropin B (Fig. 1C, D) represented the upper limit of activity among the linear peptides. By contrast, Cecropin A, Cecropin P1, the cecropin-melittin hybrid, Dermaseptin, Magainin 2, and Temporin L (Fig. 1E-H, J and K) were largely restricted to inhibition at the highest concentrations tested, and Dermaseptin-S1 (Fig. 1I) showed the clearest loss of endpoint activity with rebound growth by day 7. The two non-peptidic reference compounds, florfenicol and chlorhexidine (Fig. 1L, M), provided internal benchmarks for ribosome-targeting and membrane-active mechanisms.

Molecular dynamics reveals distinct adsorption modes on the PE-biased membrane.

**Fig. 2:**
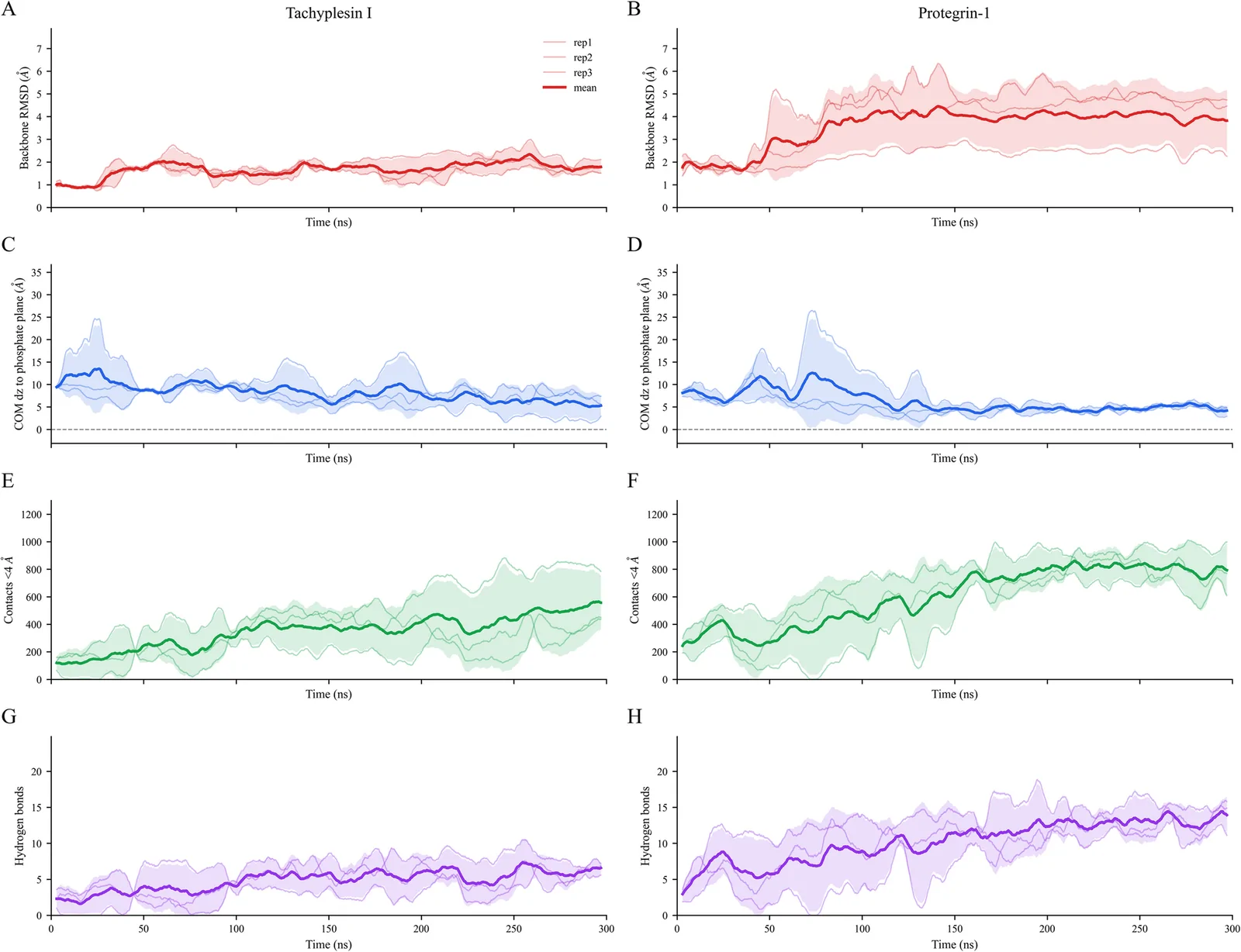
Time-resolved observables for Tachyplesin I and Protegrin-1 on the PE-biased membrane across three independent 300 ns production trajectories per peptide. Thin colored traces show the 60-frame running mean for each replicate, thick traces show the replicate mean, and shaded bands show ± SD across the three replicate trajectories. Y-axis limits are shared within each metric row to enable direct visual comparison between peptides. RMSD, root-mean-square deviation; COM, center of mass; H-bonds, hydrogen bonds; ns, nanoseconds. **A** Tachyplesin I backbone RMSD. **B** Protegrin-1 backbone RMSD. **C** Tachyplesin I signed COM z-displacement relative to the upper-leaflet phosphate plane. **D** Protegrin-1 signed COM z-displacement relative to the upper-leaflet phosphate plane. **E** Tachyplesin I peptide-lipid contacts within 4 Å. **F** Protegrin-1 peptide-lipid contacts within 4 Å. **G** Tachyplesin I peptide-lipid H-bonds. **H** Protegrin-1 peptide-lipid H-bonds

Across three 300 ns trajectories per peptide, both peptides sampled membrane-bound states within the simulated window but showed different replicate-supported interfacial behavior (Fig. 2). Tachyplesin I remained more conformationally compact, with a last-50-ns backbone RMSD of 1.88 ± 0.34 Å across replicate means, whereas Protegrin-1 showed greater conformational rearrangement, with a corresponding RMSD of 3.96 ± 1.32 Å. Protegrin-1 maintained a more consistent late-stage distance from the phosphate plane and formed more peptide-lipid contacts and H-bonds, whereas Tachyplesin I showed greater replicate-to-replicate variability in membrane proximity and contact number. These results support distinct monomeric adsorption behavior but also show that adsorption strength alone does not explain the experimental growth-inhibition ranking.

The recalculated molecular dynamics descriptors obtained from the 300-ns trajectories are presented in Table 2.

**Table 2:** Recalculated molecular dynamics descriptors for Tachyplesin I and Protegrin-1 on the PE-biased membrane. RMSD, root-mean-square deviation; IC80 and IC90, concentrations required for 80% and 90% inhibition of net OD600 growth, respectively; COM, center of mass; signed COM z-displacement is measured relative to the upper-leaflet phosphate plane

Descriptor	Tachyplesin I	Protegrin-1
IC80, µg/mL (µM)	2 (0.9)	32 (14.8)
IC90, µg/mL (µM)	16 (7.1)	32 (14.8)
Production trajectories analyzed	3 × 300 ns	3 × 300 ns
Frames analyzed	9000	9000
Peptide backbone RMSD, last 50 ns (Å)	1.88 ± 0.34	3.96 ± 1.32
Peptide signed COM z-displacement relative to phosphate plane, last 50 ns (Å)	5.98 ± 3.02	4.94 ± 0.03
Peptide-lipid contacts within 4 Å, last 50 ns	507 ± 263	800 ± 152
Peptide-lipid H-bonds, last 50 ns	6.3 ± 1.4	13.2 ± 1.9
Minimum signed COM z-displacement, range across trajectories	-0.37 to 3.37 Å	-0.25 to 1.33 Å
Maximum contact count, range across trajectories	601 to 1005	913 to 1203
Representative late-stage snapshot shown in Fig. 3	257.0 ns	272.9 ns
Representative membrane interaction shown in Fig. 3	Central-loop insertion in the representative original trajectory	N- and C-terminal anchoring in the representative original trajectory

For MD observables, values reported as mean ± SD describe the mean and standard deviation across the three independent trajectory-specific last-50-ns means, not frame-to-frame variation from a single trajectory

The quantitative comparison in Table 2 summarizes these replicate-level differences. During the last 50 ns, Tachyplesin I showed a lower backbone RMSD than Protegrin-1 (1.88 ± 0.34 Å versus 3.96 ± 1.32 Å across replicate means), consistent with greater conformational stability of the Tachyplesin I scaffold. Protegrin-1 nevertheless showed more consistent membrane proximity (4.94 ± 0.03 Å versus 5.98 ± 3.02 Å for Tachyplesin I), more peptide-lipid contacts (800 ± 152 versus 507 ± 263), and more peptide-lipid H-bonds (13.2 ± 1.9 versus 6.3 ± 1.4). Thus, the lower experimental IC80/IC90 endpoints of Tachyplesin I did not map monotonically onto simple monomeric adsorption, contact, or hydrogen-bond counts.

**Fig. 3:**
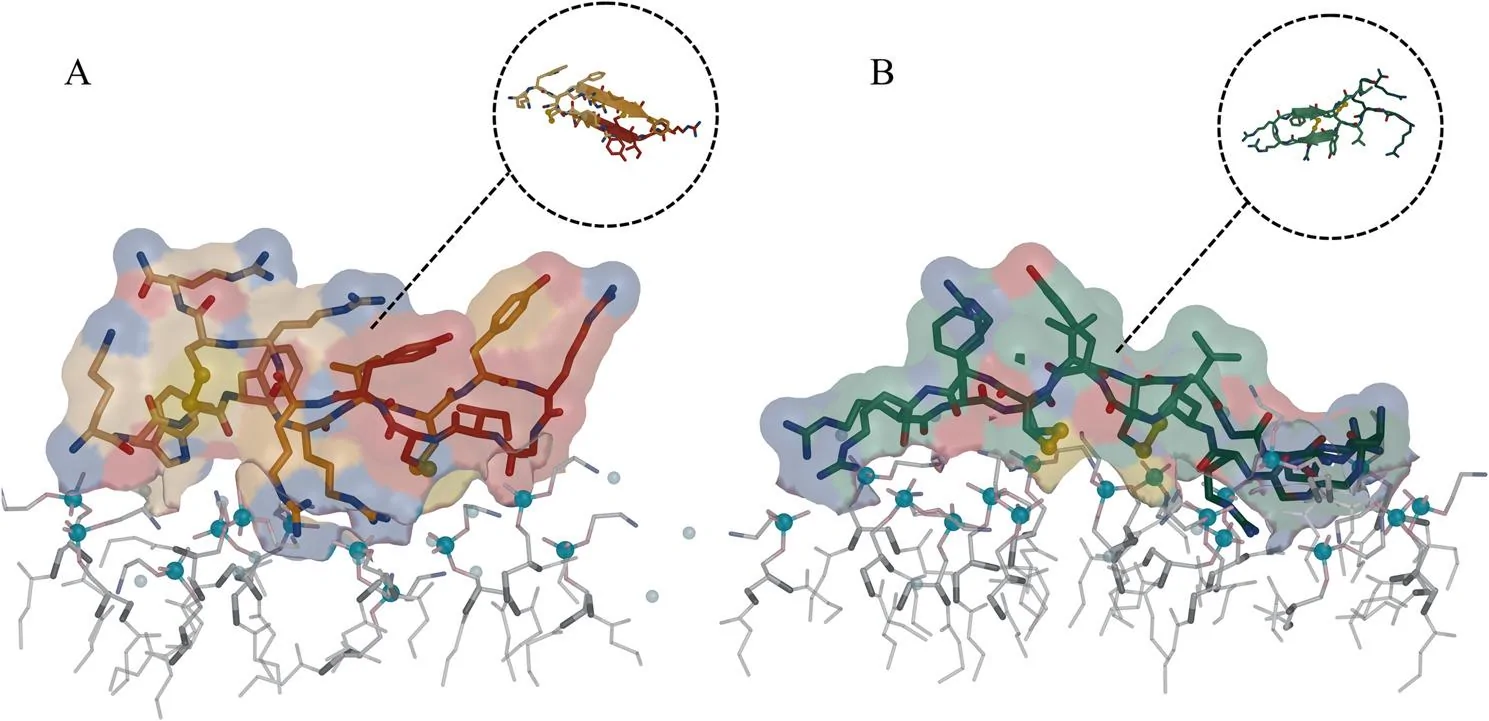
Representative late-stage membrane-bound configurations of Tachyplesin I and Protegrin-1. Representative structures were selected from the last 50 ns of each trajectory as the peptide heavy-atom conformations with minimum RMSD to the corresponding window mean. **A** Tachyplesin I at 257.0 ns. **B** Protegrin-1 at 272.9 ns. Main panels show peptide-centered side views of the peptide and nearby upper-leaflet lipids, and insets show the isolated peptide conformation from the same snapshot. Full side-view setup panels for the initial and late frames are provided as supplementary material. Peptides are shown as sticks with a semi-transparent molecular envelope. Carbon atoms are shown in orange for Tachyplesin I and green for Protegrin-1; nitrogen, oxygen, and sulfur atoms are shown in blue, red, and yellow, respectively. Lipid phosphorus atoms are shown as cyan spheres and nearby lipid atoms as gray sticks. The semi-transparent peptide surface indicates the molecular envelope and is not an electrostatic potential map

The representative late-stage configurations shown in Fig. 3 provide a structural view of the late adsorbed states summarized in Table 2. In Tachyplesin I (Fig. 3A), the peptide remains compact and approaches the upper leaflet with its central segment positioned closest to the phosphate plane, consistent with the central-loop insertion mode inferred from the trajectory. In Protegrin-1 (Fig. 3B), the peptide adopts a more laterally extended interfacial state and remains engaged with the membrane predominantly through its terminal regions, consistent with the N- and C-terminal anchoring assigned from the time-resolved analysis. These panels are intended as representative views of the late adsorbed regime rather than unique bound states.

The residue-level insertion-depth analysis underlying this interpretation is provided explicitly in Supplementary Figure S1 and Supplementary Table S1. At the final analyzed frame, Tachyplesin I placed its central residues ARG9, GLY10, ILE11, and CYS12 at or below the upper-leaflet phosphate plane, whereas both termini remained solvent exposed. In contrast, Protegrin-1 positioned ARG18 below the phosphate plane and kept ARG1-GLY4 near the phosphate plane, supporting a terminal anchoring pattern rather than the central-loop insertion observed for Tachyplesin I. Full system composition and simulation parameters are provided in Supplementary Table S2, and full side-view setup snapshots are provided in Supplementary Figure S2.

## Discussion

One reason to prioritize membrane-active antimicrobial peptides against *P. salmonis* is that they are not designed to inhibit a single intracellular protein target. Conventional antibiotics often depend on a defined molecular target and can therefore lose activity through target mutation, target protection, or drug inactivation, whereas many cationic AMPs act through multistep interactions with the bacterial envelope, including electrostatic adsorption, interfacial insertion, membrane permeabilization, and downstream disruption of envelope integrity [7–9]. This does not mean that resistance to AMPs cannot emerge, but it does provide a rationale for evaluating membrane-active scaffolds as potential complements or alternatives in a pathogen that remains strongly linked to antimicrobial use in Chilean aquaculture [3–5].

The present screen indicates that, within this 13-compound panel, peptide scaffold was associated with antimicrobial performance against *P. salmonis* under prolonged incubation at 16.5 °C. The two beta-hairpin peptides, Tachyplesin I and Protegrin-1, were the most active peptides tested, whereas florfenicol and chlorhexidine remained the most potent non-peptidic reference compounds in the full panel.

This peptide ordering is directionally consistent with the broader AMP literature, although the comparative evidence comes largely from other Gram-negative systems. Tachyplesin I has retained antimicrobial activity across structural analog series, with loss of the disulfide-stabilized beta-sheet reducing activity [31], and Protegrin-1 has repeatedly shown broad-spectrum low-µg/mL activity against representative Gram-negative bacteria [32]. Among the linear peptides in the present panel, SMAP-29 was the strongest, which is likewise consistent with prior reports that it is a potent broad-spectrum cathelicidin [33]. Cecropin B has also been investigated against aquaculture-relevant Gram-negative pathogens, but inducible adaptation has been demonstrated in several fish bacterial species [34]. Read conservatively, the present data therefore place *P. salmonis* within a known AMP response landscape rather than establishing an entirely unexpected hierarchy.

The two non-peptidic reference compounds also behaved in a way that is interpretable in the context of previous work. Florfenicol served as the therapeutic comparator expected for *P. salmonis*, and low MIC distributions have been documented for susceptible isolates under validated broth microdilution conditions [6, 35]. In the present study, however, florfenicol was analyzed with the same OD600-based IC80/IC90 endpoint used for the peptide screen, so these values should not be treated as standardized clinical MICs. Chlorhexidine, by contrast, functioned here as a membrane-active antiseptic benchmark rather than as a candidate systemic therapy. Its strong in vitro inhibition is compatible with its known ability to destabilize bacterial membranes and impair membrane-associated functions [36].

The most conservative interpretation is that this peptide ranking reflects how different AMP scaffolds interact with the unusual envelope context proposed for *P. salmonis*, rather than scaffold alone acting as a universal determinant of activity. Genome-scale reconstruction of LF-89 identified missing cardiolipin and phosphatidylglycerol biosynthetic capacity, which motivated the PE-biased membrane model used here [13], and structural analysis of *P. salmonis* lipid A identified Ara4N substitutions [15], a modification known to reduce outer-membrane negative charge and lower susceptibility to cationic compounds [16]. In that setting, linear alpha-helical peptides that often benefit from stronger interactions with more anionic bilayers may be less favored than disulfide-constrained beta-hairpin scaffolds that can retain membrane activity with limited conformational reorganization [11, 12, 37]. This explanation nevertheless remains provisional because membrane composition in the present study was inferred from genomic and biochemical evidence rather than measured directly under the assay conditions, and because the linear peptide set also differed in sequence, charge, and hydrophobicity.

The MD analysis adds mechanistic context but should be read as support for the experimental ranking, not as a direct explanation of the growth-inhibition endpoints. Both peptides adsorbed to the PE-biased bilayer, yet they reached that state through clearly different trajectories. Tachyplesin I remained structurally compact and converged to a closer membrane-bound state, whereas Protegrin-1 adsorbed earlier and accumulated more contacts and hydrogen bonds while remaining farther from the phosphate plane on average. These differences are compatible with previous biophysical work showing that tachyplesin can permeabilize membranes while retaining a constrained beta-hairpin architecture [12, 37], whereas protegrin more readily populates pore-associated and oligomer-dependent membrane states rather than a single dominant monomeric topology [38, 39]. The representative late-stage configurations in Fig. 3 are consistent with this distinction and help explain why higher interfacial contact counts did not translate directly into lower IC80/IC90 values.

This distinction is important when interpreting the relationship between adsorption metrics and growth-inhibition activity. The replicate simulations reinforce that monomeric adsorption strength, contact counts, hydrogen-bond counts, and antimicrobial growth inhibition should not be treated as interchangeable descriptors: Protegrin-1 reproducibly formed more contacts and H-bonds, whereas Tachyplesin I showed lower IC80/IC90 endpoints and a lower average RMSD. A reasonable interpretation is that Tachyplesin I may benefit from structural or membrane-disruptive features not captured by simple monomeric contact counts, whereas Protegrin-1 may depend more strongly on peptide-peptide cooperativity or pore-maturation steps that were not accessible in the present single-peptide simulations. That interpretation is consistent with prior experimental and computational studies showing that protegrin pore formation depends strongly on oligomeric organization and can give rise to several structurally distinct membrane defects [38, 39].

Several limitations define the scope of the current conclusions. First, the antimicrobial analysis was performed on a single LF-89 strain in one assay framework, whereas published susceptibility profiling shows that *P. salmonis* field isolates can differ in antibiotic response [6]. The experiment nevertheless included three independent biological replicates, each initiated from a separately seeded culture of the ATCC strain. Second, the membrane model was derived from genomic and biochemical inference rather than direct lipidomics of the same strain under the same culture conditions [13, 15], and it did not include an explicit outer membrane or lipopolysaccharide layer even though those features are likely relevant to AMP access and susceptibility in *P. salmonis* [15–17]. Third, the simulations were intentionally limited to the early monomeric adsorption stage and to single-peptide systems. We added two independently reseeded 300 ns production trajectories per peptide, giving three 300 ns trajectories per peptide in the revised analysis; however, these simulations still do not sample peptide oligomerization, pore stabilization, membrane rupture, or downstream loss of viability [38, 39]. We also did not perform circular dichroism or other post-binding structural assays, nor did we compute PMF, MM-PBSA, or other binding free-energy estimates. Finally, the present study does not address peptide stability in medium, host-cell toxicity, hemolysis, serum stability, or intracellular activity, all of which are relevant for evaluating translational potential against an intracellular fish pathogen. Even with those constraints, the convergence between the experimental ranking and the replicate-supported membrane-interaction analysis supports beta-hairpin peptides, and particularly Tachyplesin I, as the strongest peptide leads emerging from this panel and as logical candidates for broader strain testing and downstream selectivity studies.

## Conclusions

A 13-compound screen against *P. salmonis* identified Tachyplesin I and Protegrin-1 as the most active peptides tested, with both beta-hairpin scaffolds showing lower IC80/IC90 growth-inhibition endpoints than the linear peptides included in the panel. Replicate 300 ns molecular dynamics simulations showed that both leads engage the PE-biased membrane through distinct monomeric adsorption behavior: Tachyplesin I remained more conformationally compact, whereas Protegrin-1 formed more contacts and hydrogen bonds. Together, these results support further evaluation of beta-hairpin antimicrobial peptides as membrane-active leads against *P. salmonis*. They also provide a scaffold-informed rationale for prioritizing antimicrobial peptides against *P. salmonis* and suggest that membrane composition may contribute to susceptibility differences in this pathogen.

## Supplementary Information

Additional file 1: Supplementary Figure S1. Residue-level insertion depth for Tachyplesin I and Protegrin-1 on the PE-biased membrane.

Additional file 2: Supplementary Table S1. Residue-level insertion-depth values used to generate Supplementary Figure S1.

Additional file 3: Supplementary Figure S2. Full side-view setup snapshots for the molecular dynamics systems.

Additional file 4: Supplementary Table S2. Molecular dynamics system composition and simulation parameters for the Tachyplesin I and Protegrin-1 systems.

## Data Availability

The datasets generated and/or analysed during the current study are available from the corresponding author on reasonable request.

## References

[1] Rozas M, Enriquez R. Piscirickettsiosis and *Piscirickettsia salmonis* in fish: a review. J Fish Dis. 2014;37:163–88. 24279295 10.1111/jfd.12211

[2] Maisey K, Montero R, Christodoulides M. Vaccines for piscirickettsiosis (salmonid rickettsial septicaemia, SRS): the Chile perspective. Expert Rev Vaccines. 2017;16:215–28. 27690686 10.1080/14760584.2017.1244483

[3] Avendano-Herrera R, Mancilla M, Miranda CD. Use of antimicrobials in Chilean salmon farming: facts, myths and perspectives. Rev Aquac. 2023;15:89–111.

[4] Miranda CD, Godoy FA, Lee MR. Current status of the use of antibiotics and the antimicrobial resistance in the Chilean salmon farms. Front Microbiol. 2018;9:1284. 29967597 10.3389/fmicb.2018.01284 PMC6016283

[5] Cabello FC, Godfrey HP, Buschmann AH, Dolz HJ. Aquaculture as yet another environmental gateway to the development and globalisation of antimicrobial resistance. Lancet Infect Dis. 2016;16:e127–33. 27083976 10.1016/S1473-3099(16)00100-6

[6] Henriquez P, Kaiser M, Bohle H, Bustos P, Mancilla M. Comprehensive antibiotic susceptibility profiling of Chilean *Piscirickettsia salmonis* field isolates. J Fish Dis. 2016;39:441–8. 26660665 10.1111/jfd.12427

[7] Hancock REW, Sahl HG. Antimicrobial and host-defense peptides as new anti-infective therapeutic strategies. Nat Biotechnol. 2006;24:1551–7. 17160061 10.1038/nbt1267

[8] Zasloff M. Antimicrobial peptides of multicellular organisms. Nature. 2002;415:389–95. 11807545 10.1038/415389a

[9] Lazzaro BP, Zasloff M, Rolff J. Antimicrobial peptides: application informed by evolution. Science. 2020;368:eaau5480. 32355003 10.1126/science.aau5480 PMC8097767

[10] Wang G, Schmidt C, Li X, Wang Z. APD6: the antimicrobial peptide database is expanded to promote research and development by deploying an unprecedented information pipeline. Nucleic Acids Res. 2026;54:D363–74. 40927993 10.1093/nar/gkaf860 PMC12807733

[11] Gregory SM, Pokorny A, Almeida PFF. Magainin 2 revisited: a test of the quantitative model for the all-or-none permeabilization of phospholipid vesicles. Biophys J. 2009;96:116–31. 19134472 10.1016/j.bpj.2008.09.017 PMC2710023

[12] Doherty T, Waring AJ, Hong M. Peptide-lipid interactions of the beta-hairpin antimicrobial peptide tachyplesin and its linear derivatives from solid-state NMR. Biochim Biophys Acta. 2006;1758:1285–91. 16678119 10.1016/j.bbamem.2006.03.016

[13] Cortes MP, Mendoza SN, Travisany D, Gaete A, Siegel A, Cambiazo V, et al. Analysis of *Piscirickettsia salmonis* metabolism using genome-scale reconstruction, modeling, and testing. Front Microbiol. 2017;8:2462. 29321769 10.3389/fmicb.2017.02462 PMC5732189

[14] Raetz CRH, Dowhan W. Biosynthesis and function of phospholipids in *Escherichia coli*. J Biol Chem. 1990;265:1235–8. 2404013

[15] Vadovic P, Fodorova M, Toman R. Structural features of lipid A of *Piscirickettsia salmonis*, the etiological agent of the salmonid rickettsial septicemia. Acta Virol. 2007;51:249–59. 18197732

[16] Trent MS, Ribeiro AA, Lin S, Cotter RJ, Raetz CRH. An inner membrane enzyme in *Salmonella* and *Escherichia coli* that transfers 4-amino-4-deoxy-L-arabinose to lipid A: induction on polymyxin-resistant mutants and role of a novel lipid-linked donor. J Biol Chem. 2001;276:43122–31. 11535604 10.1074/jbc.M106961200

[17] Herrera V, Olavarria N, Saavedra J, Yuivar Y, Bustos P, Almarza O, et al. Complete lipopolysaccharide of *Piscirickettsia salmonis* is required for full virulence in the intraperitoneally challenged Atlantic salmon, *Salmo salar*, model. Front Cell Infect Microbiol. 2022;12:845661. 35372121 10.3389/fcimb.2022.845661 PMC8972169

[18] Canon-Jones H, Cortes H, Castillo-Ruiz M, Schlotterbeck T, San Martin R. *Quillaja saponaria* (Molina) extracts inhibits in vitro *Piscirickettsia salmonis* infections. Anim (Basel). 2020;10:2286. 10.3390/ani10122286 PMC7761688 33287333

[19] Beltran JF, Yanez A, Herrera-Belen L, Parraguez Contreras F, Blanco JA, Flores-Martin SN, et al. Antibiotic discovery against *Piscirickettsia salmonis* using a combined in silico and in vitro approach. Microb Pathog. 2023;180:106122. 37094756 10.1016/j.micpath.2023.106122

[20] Yanez A, Valenzuela K, Silva H, Retamales J, Romero A, Enriquez R, et al. Broth medium for the successful culture of the fish pathogen *Piscirickettsia salmonis*. Dis Aquat Org. 2012;97:197–205. 10.3354/dao02403 22422090

[21] R Core Team. R: A language and environment for statistical computing. Vienna: R Foundation for Statistical Computing. 2025. Available from: https://www.R-project.org/

[22] Mizuguchi M, Kamata S, Kawabata S, Fujitani N, Kawano K. Solution structure of tachyplesin I in H2O. Protein Data Bank. 2005. 10.2210/pdb1WO0/pdb.

[23] Mani R, Tang M, Wu X, Buffy JJ, Waring AJ, Sherman MA, et al. Membrane-bound dimer structure of a beta-hairpin antimicrobial peptide from rotational-echo double-resonance solid-state NMR. Biochemistry. 2006;45:8341–9. 16819833 10.1021/bi060305b

[24] Jo S, Lim JB, Klauda JB, Im W. CHARMM-GUI Membrane Builder for mixed bilayers and its application to yeast membranes. Biophys J. 2009;97:50–8. 19580743 10.1016/j.bpj.2009.04.013 PMC2711372

[25] Huang J, Rauscher S, Nawrocki G, Ran T, Feig M, de Groot BL, et al. CHARMM36m: an improved force field for folded and intrinsically disordered proteins. Nat Methods. 2017;14:71–3. 27819658 10.1038/nmeth.4067 PMC5199616

[26] Klauda JB, Venable RM, Freites JA, O’Connor JW, Tobias DJ, Mondragon-Ramirez C, et al. Update of the CHARMM all-atom additive force field for lipids: validation on six lipid types. J Phys Chem B. 2010;114:7830–43. 20496934 10.1021/jp101759q PMC2922408

[27] Hopkins CW, Le Grand S, Walker RC, Roitberg AE. Long-time-step molecular dynamics through hydrogen mass repartitioning. J Chem Theory Comput. 2015;11:1864–74. 26574392 10.1021/ct5010406

[28] Eastman P, Swails J, Chodera JD, McGibbon RT, Zhao Y, Beauchamp KA, et al. OpenMM 7: rapid development of high performance algorithms for molecular dynamics. PLoS Comput Biol. 2017;13:e1005659. 28746339 10.1371/journal.pcbi.1005659 PMC5549999

[29] Michaud-Agrawal N, Denning EJ, Woolf TB, Beckstein O. MDAnalysis: a toolkit for the analysis of molecular dynamics simulations. J Comput Chem. 2011;32:2319–27. 21500218 10.1002/jcc.21787 PMC3144279

[30] Gowers RJ, Linke M, Barnoud J, Reddy TJE, Melo MN, Seyler SL, et al. MDAnalysis: a Python package for the rapid analysis of molecular dynamics simulations. In: Benthall S, Rostrup S, editors. Proceedings of the 15th Python in Science Conference. Austin (TX): SciPy; 2016. p. 102–109.

[31] Tamamura H, Ikoma R, Niwa M, Funakoshi S, Murakami T, Fujii N. Antimicrobial activity and conformation of tachyplesin I and its analogs. Chem Pharm Bull (Tokyo). 1993;41:978–80. 8339345 10.1248/cpb.41.978

[32] Steinberg DA, Hurst MA, Fujii CA, Kung AHC, Ho JF, Cheng FC, et al. Protegrin-1: a broad-spectrum, rapidly microbicidal peptide with in vivo activity. Antimicrob Agents Chemother. 1997;41:1738–42. 9257752 10.1128/aac.41.8.1738 PMC163996

[33] Skerlavaj B, Benincasa M, Risso A, Zanetti M, Gennaro R. SMAP-29: a potent antibacterial and antifungal peptide from sheep leukocytes. FEBS Lett. 1999;463:58–62. 10601638 10.1016/s0014-5793(99)01600-2

[34] Sallum UW, Chen TT. Inducible resistance of fish bacterial pathogens to the antimicrobial peptide cecropin B. Antimicrob Agents Chemother. 2008;52:3006–12. 18474580 10.1128/AAC.00023-08 PMC2533505

[35] Contreras-Lynch S, Smith P, Olmos P, Loy ME, Finnegan W, Miranda CD. A novel and validated protocol for performing MIC tests to determine the susceptibility of *Piscirickettsia salmonis* isolates to florfenicol and oxytetracycline. Front Microbiol. 2017;8:1255. 28729865 10.3389/fmicb.2017.01255 PMC5498515

[36] Barrett-Bee K, Newboult L, Edwards S. The membrane destabilising action of the antibacterial agent chlorhexidine. FEMS Microbiol Lett. 1994;119:249–53. 8039666 10.1111/j.1574-6968.1994.tb06896.x

[37] Matsuzaki K, Yoneyama S, Fujii N, Miyajima K, Yamada K, Kirino Y, et al. Membrane permeabilization mechanisms of a cyclic antimicrobial peptide, tachyplesin I, and its linear analog. Biochemistry. 1997;36:9799–806. 9245412 10.1021/bi970588v

[38] Lazaridis T, He Y, Prieto L. Membrane interactions and pore formation by the antimicrobial peptide protegrin. Biophys J. 2013;104:633–42. 23442914 10.1016/j.bpj.2012.12.038 PMC3566446

[39] Lipkin R, Pino-Angeles A, Lazaridis T. Transmembrane pore structures of beta-hairpin antimicrobial peptides by all-atom simulations. J Phys Chem B. 2017;121:9126–40. 28879767 10.1021/acs.jpcb.7b06591 PMC5686775

